# Genome-wide analysis and expression profiling of *CPK* gene families in *Sorghum bicolor* (L.) Moench reveal their hormone-mediated responses during seed germination

**DOI:** 10.3389/fpls.2026.1897022

**Published:** 2026-07-29

**Authors:** Yuwen Jiang, Manjing Chen, Jiaqi Shen, Guobing Zhang, Qiang Zhao, Lingbi Zhou, Yan Ren, Mingbo Shao

**Affiliations:** Institute of Dryland Crops, Guizhou Academy of Agricultural Sciences, Guiyang, China

**Keywords:** bioinformatics, CPK, expression profiling, seed germination, sorghum

## Abstract

**Introduction:**

Sorghum (*Sorghum bicolor* (L.) Moench) is an economically vital crop worldwide for food, forage, and bioenergy production, and also serves as the core raw material for Guizhou-flavored Chinese Baijiu. Pre-harvest sprouting (PHS) severely impairs sorghum grain yield, quality, and industrial economic value. Calcium-dependent protein kinases (CPKs/CDPKs) are plant-specific calcium sensors that exert essential regulatory functions in seed development, dormancy, and germination. Nevertheless, the genome-wide characteristics of the sorghum SbCPK family and their molecular mechanisms underlying seed germination regulation remain largely elusive.

**Methods:**

In this study, we systematically identified the *SbCPK* gene family based on the BTx623 v2.1 reference genome, and comprehensively analyzed their physicochemical properties, phylogenetic relationships, gene structures, conserved motifs, chromosomal distribution, collinearity, and promoter cis-acting elements. Using the sorghum cultivar `Hongyingzi' as experimental material, we integrated transcriptome profiling and qRT-PCR assays to characterize the tissue-specific expression patterns of *SbCPKs* and their transcriptional responses to exogenous ABA and ACC (ethylene precursor) treatments

**Results:**

A total of 30 *SbCPK* genes were identified across the sorghum genome, which were unevenly distributed on 10 chromosomes and formed 8 tandem duplication clusters. Phylogenetic analysis classified these SbCPKs into four distinct subfamilies, which exhibited high evolutionary conservation with CPK homologs in maize and rice. Gene structures and motif compositions were conserved among the four subfamilies, and abundant cis-elements associated with ABA, ethylene, stress responses and seed development were detected in the promoters of SbCPK genes. Tissue expression profiling revealed that *SbCPK5*, *SbCPK9*, *SbCPK11*, and *SbCPK19* were specifically and highly expressed in sorghum embryos. Exogenous ABA significantly inhibited seed germination and upregulated the transcript levels of *SbCPK6*, *SbCPK8*, *SbCPK9*, *SbCPK11*, and *SbCPK21*. In contrast, ACC markedly promoted seed germination, specifically induced the expression of *SbCPK19*, and repressed the expression of multiple other *SbCPK* members. These results indicate that SbCPK family genes exhibit obvious differential expression in response to ABA and ethylene signals during sorghum seed germination.

**Discussion:**

Collectively, this study identifies key candidate *SbCPK* genes associated with seed germination and hormonal signal transduction, providing a fundamental theoretical basis for further elucidating the calcium signaling-mediated molecular regulatory mechanisms of PHS in sorghum.

## 1 Introduction

Sorghum (*Sorghum bicolor* (L.) Moench) is the world’s fifth-largest cereal crop and an important crop for food, forage, and bioenergy production (Li et al., 2026). In China, sorghum acts as the core raw material for the Chinese Baijiu industry, and the ‘Hongyingzi’ cultivar supplies over 80% of the raw materials used for high-end Baijiu manufacturing (Ding et al., 2024). Pre-harvest sprouting (PHS) defined as premature grain germination on maternal plants under high temperature and high humidity (Xu et al., 2026), has become a major constraint restricting sorghum yield and grain quality (Ju et al., 2025). PHS not only induces massive yield losses, which can reach 30%–40% in severely affected years, but also seriously deteriorates grain quality (Benech-Arnold and Rodriguez, 2018).

Seed dormancy and germination are precisely modulated by an intricate signaling network composed of phytohormones including abscisic acid (ABA) and ethylene (ETH), which exert an antagonistic regulatory relationship in gramineous crops. ABA maintains seed dormancy and impedes radicle protrusion by activating the ABI3-ABI5 signaling cascade. In contrast, ethylene functions via the EIN2-EIN3-ERF signaling axis. It alleviates ABA-mediated repression by up-regulating PP2C negative regulators (ABI1/ABI2) within the ABA pathway and accelerating ABA catabolism, thereby releasing seed dormancy and facilitating seed germination (Arc et al., 2013; An et al., 2025). This regulatory framework has been thoroughly characterized in the model plant *Arabidopsis thaliana*. Nevertheless, the ABA-ethylene crosstalk exhibits substantial inter-species divergence among gramineous crops such as rice, wheat, sorghum and maize. During imbibition-triggered wheat seed germination, ethylene merely mediates endosperm starch degradation but fails to directly break embryonic dormancy. Its antagonistic effect against ABA relies on synergistic activation of endogenous gibberellin (GA), which differs from the independent inhibitory effect of ethylene on ABA signaling in Arabidopsis (Sun et al., 2020). In rice, OsNAC2 concurrently targets the ethylene-synthetic gene *Osasco* and the ABA-biosynthetic gene *OsNCED3* to integrate ABA-ethylene signaling crosstalk and modulate germination rate, representing a monocot-specific transcriptional regulatory module (Yu et al., 2021). Exogenous ethylene down-regulates *SbABI5* transcription in sorghum, reduces seed ABA sensitivity and consequently inhibits pre-harvest sprouting (Rodríguez et al., 2009). Nevertheless, the molecular mechanism underlying ABA-ethylene crosstalk governing sorghum seed germination remains largely underexplored. Deciphering how ABA-ethylene interplay modulates seed dormancy-germancy events provides critical theoretical support for breeding sorghum cultivars with enhanced pre-harvest sprouting resistance (Dong and Wang, 2024).

Calcium ions (Ca^2+^) are ubiquitous second messengers in eukaryotic cells, mediating various cellular processes such as cell division, transcriptional regulation, stomatal movement, and responses to biotic and abiotic stresses (Harper and Harmon, 2005; Schulz et al., 2013). Calcium-dependent protein kinases (*CDPK*/*CPKs*) are plant-specific Ca^2+^ sensor. Their structure consists of an N-terminal variable domain, a conserved serine/threonine kinase domain, an autoinhibitory domain, and a C-terminal calmodulin-like domain containing an EF-hand Ca*^2+^*-binding motif (Colbran et al., 1989). Upon sensing changes in intracellular Ca^2+^concentrations induced by environmental or developmental signals, *CPK* undergoes conformational changes, releases autoinhibition, and phosphorylates downstream target proteins, thereby amplifying and transducing stress signals to activate adaptive physiological and biochemical responses (Kudla et al., 2010).

Accumulating studies have demonstrated that the CPK gene-family performs vital functions in plant abiotic-stress responses and seed-development processes (Delormel and Boudsocq, 2019). 34 *CPKs* have been identified in *A. thaliana*. Among them, AtCPK6 and AtCPK32 phosphorylate ABA-responsive ABF transcription factors to positively activate ABA signaling, thereby regulating seed germination and improving drought-stress tolerance (Cheng et al., 2002). Within gramineous cereal crops, 84, 39 and 31 CPK family members have been characterized in wheat, maize and rice genomes, respectively (Liu et al., 2023; Weckwerth et al., 2014; Ray et al., 2007; Li et al., 2008). Previous functional investigations on gramineous-origin CPKs have predominantly focused on abiotic-stress adaptations including drought, salinity and cold resistance. These CPKs confer stress tolerance by activating antioxidant systems, maintaining cellular osmotic homeostasis and stabilizing cell-membrane structures. Recent evidence further reveals that CPKs act as core hubs mediating ABA-ethylene hormonal crosstalk in gramineous crops, modulating seed dormancy-germination as well as PHS. In wheat, 11 *TaCPKs* exhibit differential expression during seed dormancy and germination, and TaCPK40 accelerates seed germination by negatively regulating ABA signaling (Liu et al., 2023). Rice OsCPK29 phosphorylates the ABI5 protein; it represses ABA-dependent signaling to facilitate seed germination and meanwhile responds to ethylene signals to precisely fine-tune seed-dormancy levels (Liang et al., 2025). Maize ZmCPK11 integrates the ABA signaling cascade and is transcriptionally induced by ethylene, which suppresses precocious kernel germination at the late grain-development stage (Ding et al., 2013). These findings indicate that CPK homologs governing seed-germination programs have evolved divergent regulatory roles across different gramineous species. Beyond *Gramineae*, 17, 30, 30, 29 and 28 CPK genes have been separately identified in pineapple, moso-bamboo, rubber tree, tomato and barley (Zhang et al., 2020; Wu et al., 2023; Xiao et al., 2016; Wang et al., 2016; Zhang et al., 2015). Although the stress-related functions of CPKs have been documented in these plant species, the regulatory roles of sorghum *SbCPKs* in ABA-ethylene-dependent seed germination and pre-harvest sprouting remain largely uncharacterized.

Although the *CPK* gene family has been extensively characterized in model plants and major cereal crops, research regarding the sorghum *CPK* gene family remains limited (Dekomah et al., 2022). Existing studies on sorghum *CPKs* have mainly focused on the functional validation of individual genes under specific stress conditions, while comprehensive genome-wide identification and systematic characterization based on the sorghum reference genome are still insufficient (Fan et al., 2023). Notably, the expression patterns of sorghum *CPK* genes during seed germination and their transcriptional responses to germination-related phytohormones (e.g., ABA and ethylene) remain uncharacterized.

In this study, we performed genome-wide identification of the *CPK* gene family based on the BTx623 v2.1 reference genome, followed by systematic analyses of physicochemical properties, phylogenetic relationships, gene structures, conserved motifs, chromosomal localization, gene duplication events, and *cis*-acting regulatory elements. Furthermore, the elite sorghum cultivar ‘Hongyingzi’ was used as plant material to investigate the tissue-specific expression profiles of *SbCPK* genes and their dynamic expression patterns during seed germination in response to exogenous ABA and 1-aminocyclopropane-1-carboxylic acid (ACC), the direct biosynthetic precursor of ethylene. The results of this study provide a preliminary theoretical basis for elucidating the molecular functions of *SbCPK* genes in sorghum seed germination and offer potential candidate genes for the molecular breeding of PHS resistant sorghum varieties.

## 2 Materials and methods

### 2.1 Identification of *SbCPK* genes

To systematically identify sorghum CPK family members, we downloaded HMM profiles of PF00069 (Pkinase) and PF13499 (EF-hand) from the Pfam database. We ran hmmscan (HMMER 3.3.2) against the sorghum reference proteome with an E-value threshold ≤1e-5 for preliminary screening (Mittal et al., 2017). Candidate sequences were further verified via SMART (http://smart.embl.de, Accessed: Feb 10, 2026) and NCBI CDD (https://www.ncbi.nlm.nih.gov/cdd, Accessed: Feb 10, 2026); only sequences carrying intact, non-truncated Pkinase and EF-hand domains were kept, while those with abnormal lengths were removed to filter false positives.

All candidate proteins were mapped to the sorghum genome annotation to obtain stable locus IDs, full CDS and protein sequences. Multiple alternative splicing isoforms were annotated for several SbCPK loci. For each gene, the longest protein isoform with complete conserved domains was chosen as the representative transcript, and all truncated, redundant isoforms were discarded to construct a non-redundant *SbCPK* dataset. All locus identifiers, full protein sequences of SbCPKs are summarized in **Supplementary Table 1**, with a table footnote explaining the isoform screening strategy and alternative transcript information. The finalized non-redundant sequences were defined as *SbCPK* gene family members (Ju et al., 2025).

### 2.2 Analysis of the physicochemical properties and chromosomal localization of the sorghum *CPK* gene family

The physicochemical properties of the amino acid sequences of members of the sorghum *CPK* gene family were estimated using the online tool ProtParam (https://web.expasy.org/protparam/, Accessed: Feb 11, 2026). Subcellular localization of SbCPK proteins was predicted online via Cell-PLoc-2 (http://www.csbio.sjtu.edu.cn/bioinf/Cell-PLoc-2/, Accessed: Feb 11, 2026), which adopts the k-nearest neighbor (k-NN) classification algorithm to predict protein subcellular locations in plant species. Gene annotation information for members of the sorghum *CPK* gene family was extracted, and chromosomal localization of the identified members was performed using TBtools v1.120 (Chen et al., 2020).

### 2.3 Phylogenetic analysis of the sorghum *CPK* gene family

CPK protein sequences for rice, maize and Arabidopsis were downloaded separately. The rice data were downloaded from the Rice Genome Annotation Project database (http://rice.uga.edu/, Accessed: Feb 11, 2026) (Hamilton et al., 2025); the maize data were downloaded from the MaizeGDB database (https://download.maizegdb.org/, Accessed: Feb 11, 2026) (Woodhouse et al., 2021); *A. thaliana* reference genome data (version TAIR10) was downloaded from the NCBI GenBank database (https://www.ncbi.nlm.nih.gov/, Accessed: Feb 11, 2026) (Lamesch et al., 2012). Multiple sequence alignment of SbCPK full-length protein sequences from the four species was performed using ClustalW in MEGA11 (version 11.0.13) (Tamura et al., 2021). The phylogenetic tree was constructed via the Neighbor-Joining (NJ) algorithm with the following parameters: Poisson correction model, pairwise deletion for gaps/missing data, and 1000 bootstrap replicates to evaluate the reliability of each clade. The tree was finally visualised using the online platform iTOL v6.7.6 (https://itol.embl.de/, Accessed: Feb 11, 2026) (Letunic and Bork, 2024).

### 2.4 Analysis of conserved motifs, conserved domains and gene structures in members of the sorghum *CPK* gene family

Conserved motifs encoded by sorghum CPK proteins were identified via the online MEME v5.5.7 tool (https://meme-suite.org/meme/tools/meme, Accessed: Feb 14, 2026), with the maximum number of detected motifs limited to 10 (Bailey et al., 2015). Gene annotation data of sorghum *CPKs* were retrieved from the whole-genome annotation file. TBtools v1.120 was subsequently applied to integrate and visualize conserved motifs, conserved domains, and gene structures (Chen et al., 2020).

Three-dimensional protein structures were predicted using the AlphaFold3 online platform (https://alphafoldserver.com/, Accessed: Feb 16, 2026), and structural visualization was performed with the Protein Viewer plugin embedded in VS code 1.99.3 software (Abramson et al., 2024).

### 2.5 Collinearity analysis

The sequence similarity of SbCPK proteins was analyzed using the EMBL-EBI online platform (https://www.ebi.ac.uk/jdispatcher, Accessed: Feb 16, 2026) (Madeire et al., 2022). Corresponding similarity heatmaps were plotted in R version 4.4.1 with the ComplexHeatmap and circlize packages (Gu et al., 2014).

The One Step MCscanX tool embedded in TBtools was employed for intra-sorghum gene collinearity analysis and interspecific collinearity comparison among sorghum, Arabidopsis, maize, and rice, followed by result visualization (Chen et al., 2020; Wang et al., 2012).

### 2.6 Analysis of *cis*-acting elements in *SbCPK* gene promoter

The 2000 bp upstream sequences of the start codons of the *SbCPK* genes were extracted as the promoter sequences via Tbtools (Chen et al., 2020). The *cis*-acting regulatory elements within these promoter regions were predicted and annotated using the PlantCARE online database (http://bioinformatics.psb.ugent.be/webtools/plantcare/html/, Accessed: Feb 14, 2026) (Lescot et al., 2002), and the resulting data were visualised with TBtools (Chen et al., 2020).

### 2.7 *In silico* expression analysis of *SbCPK* genes

Transcriptomic data for the *SbCPK* genes were downloaded from the SorghumBase database (https://www.sorghumbase.org, Accessed: Feb 16, 2026) (Gladman et al., 2026). Heatmaps of *SbCPK* gene expression in different tissues were generated using R 4.4.1 (Gu et al., 2014).

### 2.8 Plant materials and qRT-PCR analysis of gene expression

The sorghum cultivar ‘Hongyingzi’ (supplied by Maotai Group Hongyingzi Agricultural Science and Technology Development Co., Ltd.) was used as the experimental material in this study. Uniform and plump sorghum seeds with intact morphological structure were screened for subsequent germination assays. To inhibit bacterial and fungal contamination during seed germination, all selected seeds were pretreated with 0.5% Plant Preservative Mixture (Plant Cell Technology Inc., USA) solution for 2 h, followed by thorough rinsing with sterile water 3–4 times to remove residual reagent. For exogenous hormone treatments, the sterilized seeds were randomly grouped and subjected to 10 min soaking in sterile water (control), 75 μM ABA solution (Yao et al., 2025) or 20 μM ACC solution (Stockton, 1999), respectively. Subsequently, the treated seeds were placed on filter-paper-lined culture plates supplemented with 10 mL of the corresponding treatment solution for constant cultivation in a light-controlled incubator. Seed phenotypic changes were photographed at 0, 6, 12, 24, 48, and 72 h after treatment. After cultivation, the seed germination rate, seedling plumule length, and radicle length of each group were statistically measured and recorded. Meanwhile, approximately 20 seeds were sampled at each time point, rapidly frozen in liquid nitrogen, and preserved at -80°C for subsequent total RNA extraction.

Total RNA was isolated from sorghum seeds using the RNAprep Pure Polysaccharide and Polyphenol Plant Total RNA Extraction Kit (DP441, Tiangen Biotech (Beijing) Co., Ltd., Beijing, China). First-strand cDNA was synthesized via reverse transcription using the TransScript^®^ Uni All-in-One First-Strand cDNA Synthesis SuperMix for qPCR (AU341-02, TransGen Biotech, Beijing, China) with integrated genomic DNA removal. Gene-specific primers were designed with the Primer3Plus (https://www.primer3plus.com/) and commercially synthesized and purified by Sangon Biotech. All primer sequences are listed in **Table 1**. Quantitative real-time PCR (qRT-PCR) was performed on a LightCycler^®^ 96 real-time PCR system (Roche Diagnostics GmbH, Mannheim, Germany) using the PerfectStart^®^ Green qPCR SuperMix (AQ601, TransGen Biotech). The PCR program included an initial denaturation at 95 °C for 3 min, followed by 45 cycles of 95°C for 10 s and 60°C for 30 s. The sorghum *β-Actin* gene (*Sobic.001G037100*) was employed as the internal reference gene. Relative transcript expression levels were calculated based on the 2^-ΔΔCt^ method.

**Table 1:** Primer sequences used for qRT-PCR analysis.

Name	Primer sequence (5′-3′)
CPK19-Q-F	CACCTCTCCGAGCACCCAAACG
CPK19-Q-R	ATCCCTCCACGACCCCAACAAT
CPK5-Q-F	GAAGGAGAATTACAGTGAGCAG
CPK5-Q-R	GGTTTGAAGAACACAGAGAGAC
CPK9-Q-F	GTGCGCTCTGTCTACTCCCTCG
CPK9-Q-R	TCCTCCCTGTCTGCCTTGCTGG
CPK11-Q-F	CTGTCTGGTGTTCCTCCTTTTT
CPK11-Q-R	TCTGGTCGCAGCATCTTCTTAA
CPK24-Q-F	CATGAATAAACTGGAGAGGGAG
CPK24-Q-R	CAAGATGAACATCAGAAAGACC
CPK6-Q-F	GAGTGACTACTACAACCGCCCC
CPK6-Q-R	GATCCAATCCCCTCTTCAAAAT
CPK8-Q-F	AAAAAACAAGCCAGATTTCCGC
CPK8-Q-R	CCTTTCCTCCTTCTCTCACCCA
CPK29-Q-F	TATCCTCTACATCCTGCTATCC
CPK29-Q-R	TCTTGACCAAATCTTTTGCGCT
CPK21-Q-F	TCTCGCATTAAGCAATTCTCTG
CPK21-Q-R	GAACATTTCCTTCAACCCCGCA
CPK27-Q-F	AGCCCACGCTCCTGCTCCTCCT
CPK27-Q-R	CCGTCCCGAACTCCATCTCCGA
β-Actin-Q-F	CCCAAGGCTAACCGTGAGAA
β-Actin-Q-R	CGCTGGCATACAGAGAGAGG

### 2.9 Statistical analysis

Statistical analysis was performed using SPSS (version 27.0). Comparisons across three or more groups were performed via one-way analysis of variance (one-way ANOVA) and different lowercase letters (a, b, c) above the data indicate statistically significant differences between groups at the threshold of *p* < 0.05. Graphs were generated using GraphPad Prism 9.

## 3 Results and analysis

### 3.1. Identification and physicochemical property of SbCPK genes

**Figure 1:**
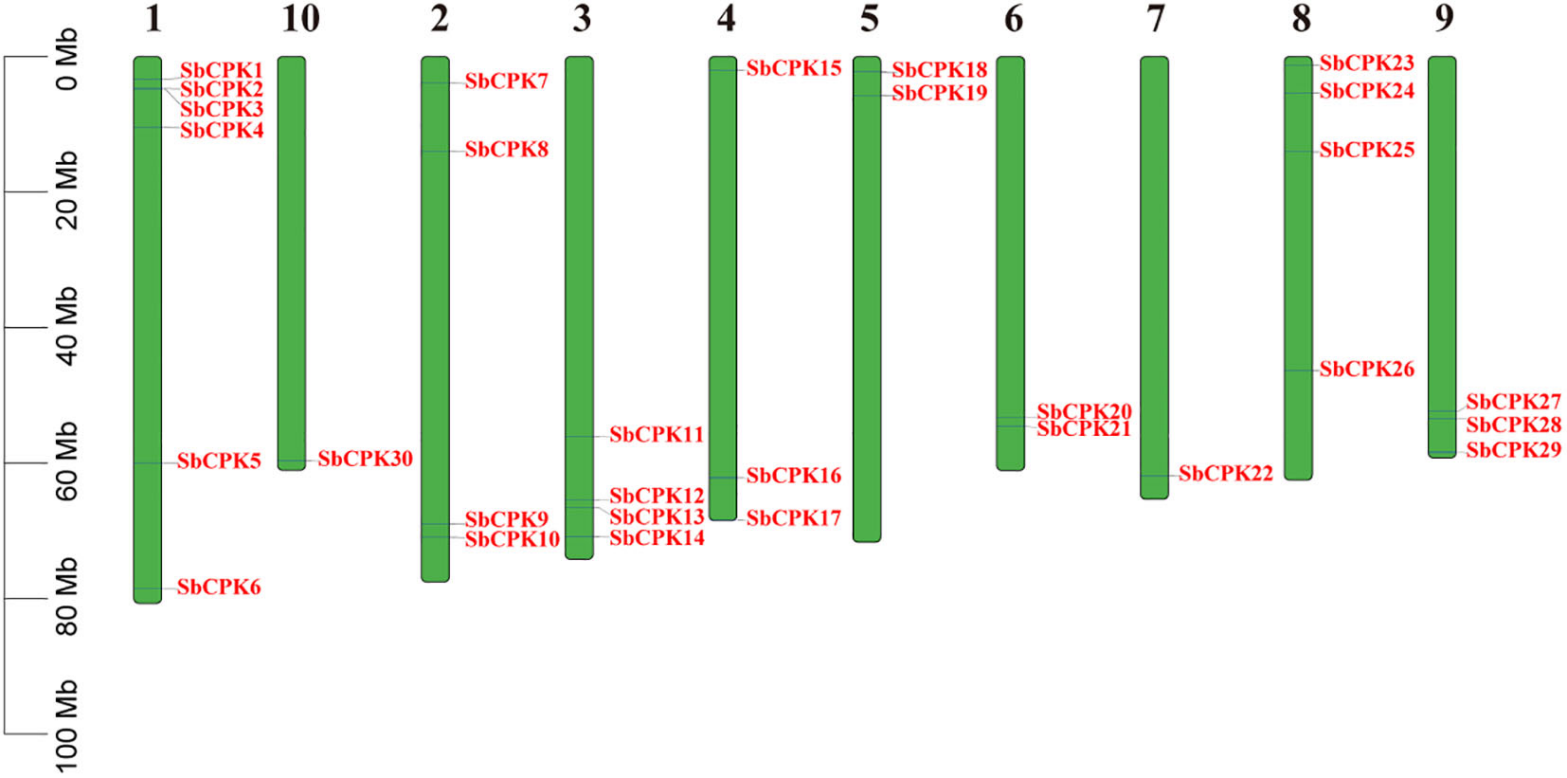
Chromosome localization of *SbCPK* genes. The chromosome numbers are indicated above each vertical bar, and the scales of the chromosomes are in megabases (Mb).

The 30 identified *SbCPK* genes were unevenly distributed across 10 chromosomes of *S. bicolor* (**Figure 1**). Chromosome 1 harbored the largest number of *SbCPK* genes (six genes), followed by chromosome 3 with five genes and chromosome 8 with four genes. Chromosomes 2, 5, and 9 each contained three *SbCPK* genes, while chromosomes 4 and 6 each possessed two *SbCPK* genes, while chromosomes 7 and 10 only carried a single *SbCPK* gene, namely *SbCPK22* and *SbCPK30*, respectively.

A total of 8 tandemly arranged gene clusters comprising 24 *SbCPK* genes were identified in the sorghum genome. These clusters included *SbCPK1*/*SbCPK2*/*SbCPK3*/*SbCPK4* on chromosome 1, *SbCPK7*/*SbCPK8*/*SbCPK9*/*SbCPK10* on chromosome 2, *SbCPK12*/*SbCPK13*/*SbCPK14* on chromosome 3, *SbCPK15*/*SbCPK16*/*SbCPK17* on chromosome 4, *SbCPK18*/*SbCPK19* on chromosome 5, *SbCPK20*/*SbCPK21* on chromosome 6, *SbCPK23*/*SbCPK24*/*SbCPK25*/*SbCPK26* on chromosome 8 and *SbCPK27*/*SbCPK28* on chromosome 9.

The length of SbCPK proteins ranged from 296 to 617 amino acids (AA), with a maximum molecular weight (MW) of 68.48 kDa (SbCPK26) and a minimum of 33.63 kDa (SbCPK22). The isoelectric point (pI) of these SbCPK proteins ranged from 4.95 to 8.90, and their grand average of hydropathicity (GRAVY) values varied from -0.605 to -0.250. The negative GRAVY values indicated that SbCPK proteins exhibit an overall hydrophilic nature at the full-protein level. Nevertheless, the presence of local hydrophobic regions and potential lipid modification sites implies that these proteins may possess partial hydrophobic characteristics, which could further affect their subcellular localization and biological behavior (**Table 2**). Subcellular localization predictions indicated that SbCPK2, 9, 12, 13, 24, 26, 28, and 30 were localized in the chloroplast; SbCPK1, 3, 4, 5, 6, 7, 8, 10, 14, 15, 17, 18, 19, 20, 23, 27, and 29 in the cytoplasm; SbCPK22 in the endoplasmic reticulum; and SbCPK11, 16, 21, and 25 in the mitochondria. Current studies have demonstrated that N-terminal myristoylation at the second-position glycine (Gly2) and subsequent palmitoylation are critical for plasma-membrane targeting of CPK proteins (Martín and Busconi, 2000; Lu and Hrabak, 2013). In this study, we further analyzed the N-terminal amino acid sequences of sorghum CPK proteins. The second-position residue in SbCPK17, SbCPK19, SbCPK22, SbCPK24 and SbCPK30 was not glycine, implying that these CPK isoforms lack the capacity for plasma-membrane anchoring (**Supplementary Table 1**). Notably, subcellular-localization prediction assigned SbCPK22 to the endoplasmic reticulum (**Table 2**). However, several SbCPKs harboring the conserved Gly2, such as SbCPK1-8, SbCPK10 and SbCPK14, were predicted to reside in the cytoplasm. These prediction patterns deviate from the canonical dual-acylation-dependent plasma-membrane-localization model. Therefore, subsequent subcellular-localization assays are required to validate the actual subcellular distribution and biological functions of sorghum CPK members.

**Table 2:** Characteristics of *SbCPKs.*.

Name	Gene ID	Chr.	AA	MW(kDa)	pI	GRAVY	Subcellular localization
SbCPK1	SORBI_3001G045000	1	532	60.00	6.36	-0.475	cytoplasm
SbCPK2	SORBI_3001G063300	1	585	64.19	5.29	-0.315	cytoplasm
SbCPK3	SORBI_3001G063600	1	617	67.53	5.95	-0.291	cytoplasm
SbCPK4	SORBI_3001G132800	1	586	64.91	8.90	-0.467	cytoplasm
SbCPK5	SORBI_3001G313100	1	538	61.04	5.01	-0.487	cytoplasm
SbCPK6	SORBI_3001G520900	1	544	61.65	5.22	-0.331	cytoplasm
SbCPK7	SORBI_3002G040800	2	581	63.95	5.63	-0.320	cytoplasm
SbCPK8	SORBI_3002G114800	2	514	57.59	7.63	-0.372	cytoplasm
SbCPK9	SORBI_3002G317300	2	531	59.52	5.93	-0.416	chloroplast
SbCPK10	SORBI_3002G343500	2	543	61.18	7.26	-0.497	cytoplasm
SbCPK11	SORBI_3003G225200	3	525	59.31	5.95	-0.530	mitochondrion
SbCPK12	SORBI_3003G329600	3	520	57.41	5.57	-0.408	chloroplast
SbCPK13	SORBI_3003G345000	3	545	60.73	6.16	-0.401	chloroplast
SbCPK14	SORBI_3003G400700	3	557	62.65	6.41	-0.535	cytoplasm
SbCPK15	SORBI_3004G025000	4	525	58.79	8.68	-0.403	cytoplasm
SbCPK16	SORBI_3004G279100	4	565	62.15	5.57	-0.276	mitochondrion
SbCPK17	SORBI_3004G358000	4	504	55.74	5.05	-0.250	cytoplasm
SbCPK18	SORBI_3005G025400	5	538	58.85	5.94	-0.403	cytoplasm
SbCPK19	SORBI_3005G056600	5	515	57.10	5.38	-0.304	cytoplasm
SbCPK20	SORBI_3006G177500	6	533	59.70	5.94	-0.442	cytoplasm
SbCPK21	SORBI_3006G192500	6	555	61.17	5.43	-0.288	mitochondrion
SbCPK22	SORBI_3007G185801	7	296	33.63	4.95	-0.483	endoplasmic reticulum
SbCPK23	SORBI_3008G015600	8	574	62.54	5.88	-0.394	cytoplasm
SbCPK24	SORBI_3008G053300	8	515	56.73	5.41	-0.286	chloroplast
SbCPK25	SORBI_3008G080700	8	569	63.27	6.25	-0.369	mitochondrion
SbCPK26	SORBI_3008G097800	8	616	68.48	5.48	-0.468	chloroplast
SbCPK27	SORBI_3009G167900	9	543	60.46	6.33	-0.412	cytoplasm
SbCPK28	SORBI_3009G180600	9	527	58.06	5.61	-0.383	chloroplast
SbCPK29	SORBI_3009G249000	9	541	61.09	6.44	-0.605	mitochondrion
SbCPK30	SORBI_3010G260600	10	516	57.19	5.53	-0.259	chloroplast

### 3.2 Phylogenetic analysis of SbCPK proteins

**Figure 2:**
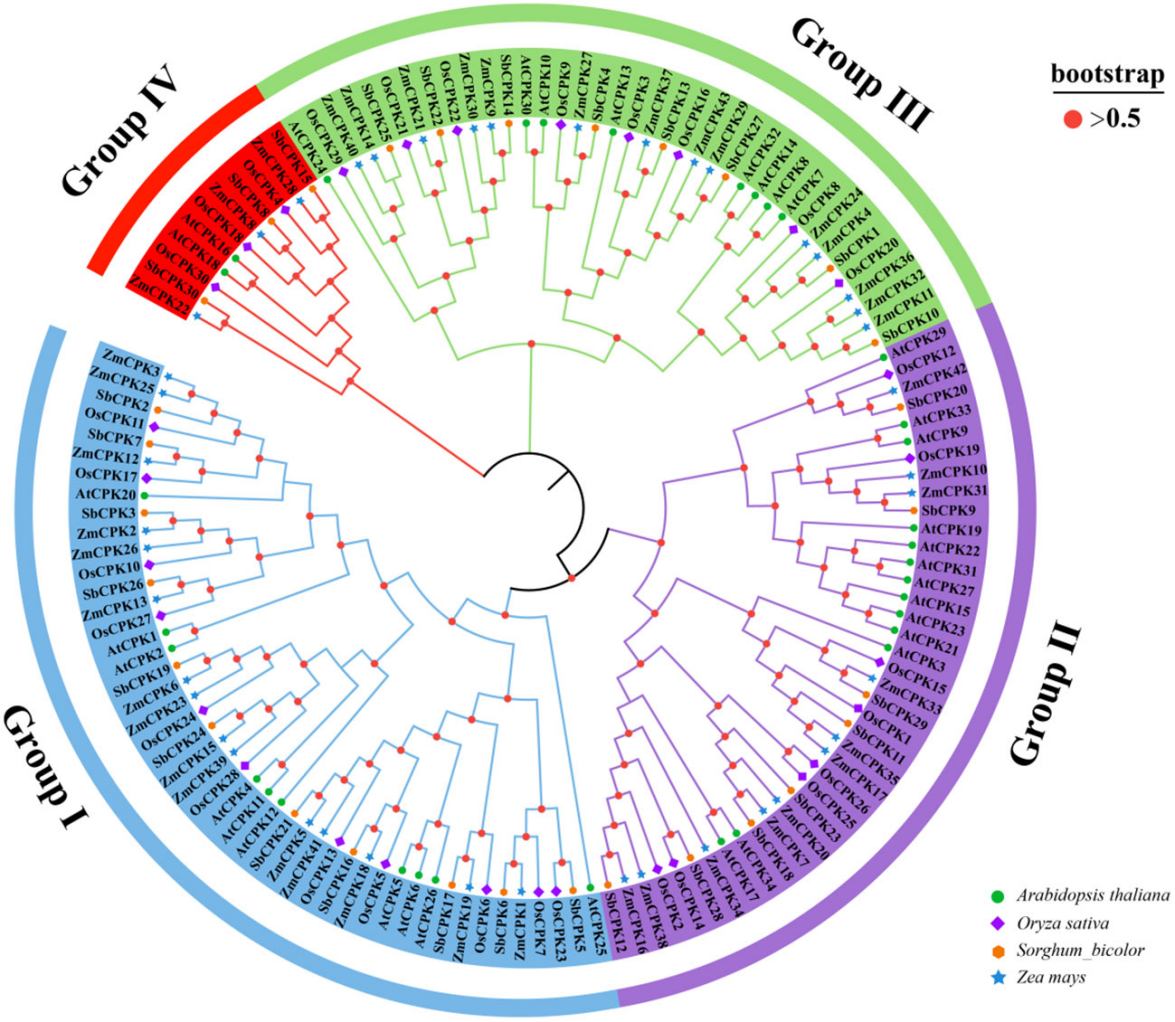
Phylogenetic analysis of SbCPK proteins across representative plant species. CPK sequences from *A. thaliana* (AtCPK), *O. sativa* (OsCPK), *Z. mays* (ZmCPK) and *S. bicolor* (SbCPK) were aligned with ClustalX 2.0, and the NJ phylogenetic tree was constructed with 1000 bootstrap replicates. Branches with bootstrap values >0.5 are marked with red dots.

A neighbor-joining phylogenetic tree was constructed based on aligned protein sequences of CPK family members from multiple plant species, including *A. thaliana* (AtCPK), *O. sativa* (OsCPK), and *Z. mays* (ZmCPK). As shown in **Figure 2**, all CPK proteins were clustered into four distinct subgroups. Group 1 harbored the largest number of members (11), while Group 2 and Group 3 each contained 8 members, and Group 4 had the fewest with only 3 members. Notably, CPK from *O. sativa*, *A. thaliana*, *Z. mays*, and *S. bicolor* were distributed across all four subgroups, indicating that the evolutionary divergence of the CPK family predated the speciation of these plant species.

### 3.3 Intron/exon organization and conserved domains analysis of *SbCPK* genes

**Figure 3:**
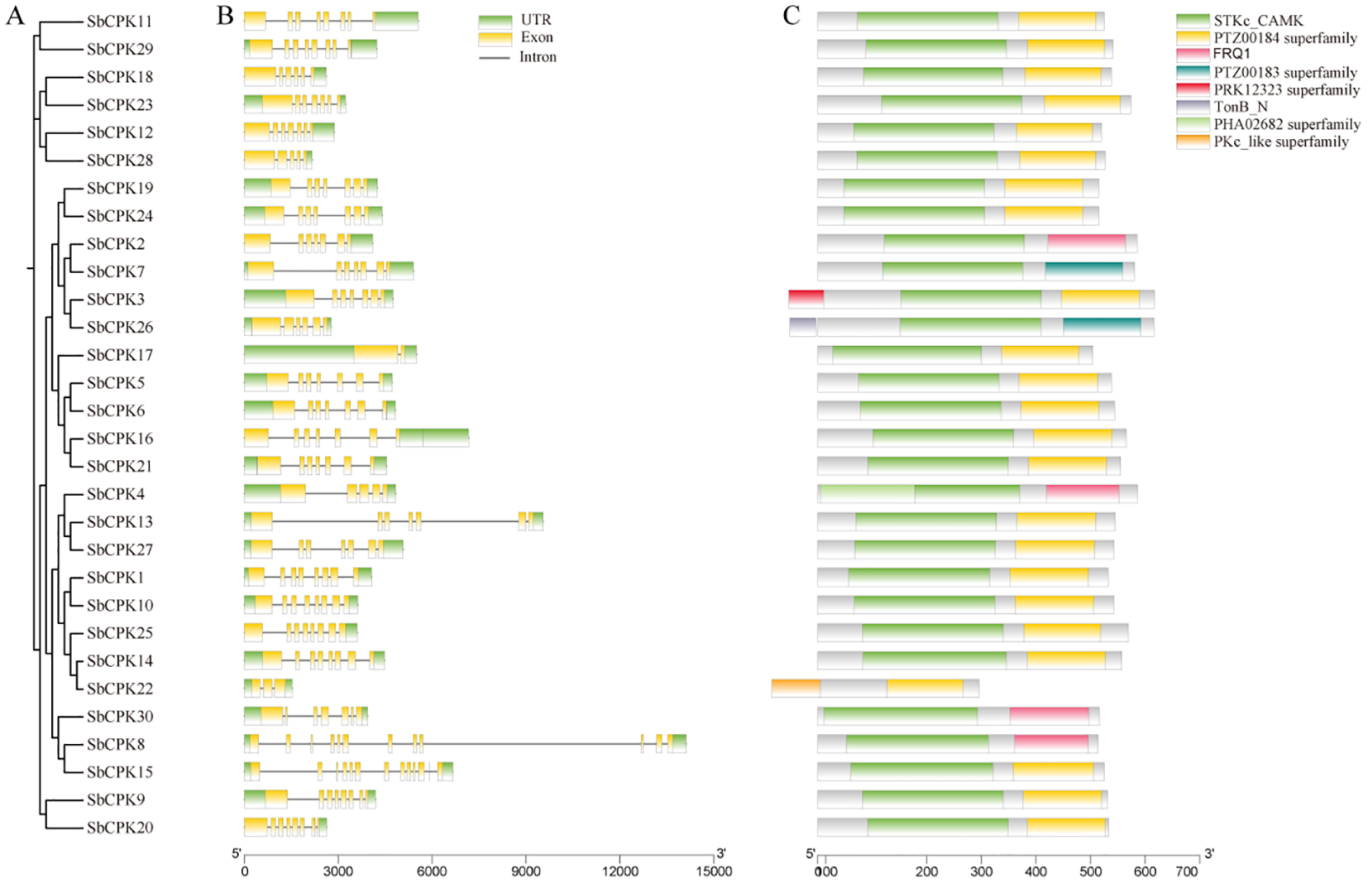
Phylogenetic relationships, conserved protein domains and gene structures of SbCPKs. **(A)** Neighbor-joining phylogenetic tree of SbCPK proteins. **(B)** Exon-intron structures of *SbCPK* genes. Green boxes: UTR; yellow boxes: CDS; black lines: introns. **(C)** Distribution of conserved domains in SbCPK proteins.

Gene structure analysis revealed that the number of exons among *SbCPK* family genes ranged from 2 to 13. *SbCPK17* possessed the fewest exons (only two; **Figure 3B**), while *SbCPK8* and *SbCPK15* contained the maximum number of 13 exons each. Intriguingly, these two genes clustered within the same phylogenetic clade (**Figure 3A, B**). Their conserved gene structures and close evolutionary relatedness implied that they might perform analogous biological functions. Moreover, genes grouped in the same clade generally exhibited identical or highly similar exon counts: for instance, both *SbCPK11* and *SbCPK29* harbored eight exons, and *SbCPK19* and *SbCPK24* each contained seven exons (**Figures 3A, B**). Conserved protein domain prediction demonstrated that all SbCPK proteins except SbCPK22 harbored a canonical STKc-CAMK (Catalytic domain of calcium/calmodulin-dependent Serine/Threonine protein kinase) domain at the N-terminus, which endows them with serine/threonine kinase catalytic activity (**Figure 3C**; **Supplementary Table 2**). The C-terminal region of most SbCPKs contained a calmodulin-like domain (PTZ00184 superfamily), characterized by four tandem EF-hand Ca^2+^-binding motifs (**Figure 3C**; **Supplementary Table 2**). This domain functions as an intracellular Ca^2+^ sensor; upon Ca^2+^ binding, it relieves the autoinhibition of the STKc-CAMK kinase domain and consequently activates the phosphorylation activity of CPK proteins (Klimecka and Muszyńska, 2007). Additionally, structural prediction indicated that SbCPK7 and SbCPK26 carried a centrin domain homologous to calmodulin at their C-termini, which was classified into the PTZ00183 superfamily. Meanwhile, the C-termini of SbCPK2, SbCPK4, SbCPK8 and SbCPK30 all possessed the conserved FQRT1 motif (**Figure 3C**). Shared conserved domains suggest that SbCPK family members transduce regulatory signals via largely conserved molecular mechanisms, whereas divergent domain architectures indicate functional differentiation among paralogous SbCPK proteins.

Protein-structure prediction revealed that all SbCPK proteins possess the canonical bipartite architecture characteristic of CPKs: a tightly-folded, conserved Serine/Threonine-kinase domain (helix-sheet regions colored in blue) and four C-terminal EF-hand Ca^2+^-binding domains. The kinase-EF-hand modular assembly is tightly interconnected, which serves as the structural foundation enabling CPKs to bind Ca^2+^ and undergo autophosphorylation independent of calmodulin. This feature confers Ca^2+^-dependent kinase catalytic activity to all SbCPK isoforms and is evolutionarily conserved. Within each subgroup, CPK paralogs exhibit highly similar overall folding patterns. For example, SbCPK11 and SbCPK29 belong to Group II; their kinase domains display nearly identical arrangements of α-helices and β-sheets, with only minor differences in the length of the disordered flexible N-terminal loop. The conserved spatial configuration of kinase domains within a given subfamily maintains conserved substrate-phosphorylation patterns, implying that these paralogs likely target a highly overlapping set of downstream substrate proteins (**Supplementary Figure 1**).

### 3.4 Identity and collinearity analysis of *SbCPK* family genes

**Figure 4:**
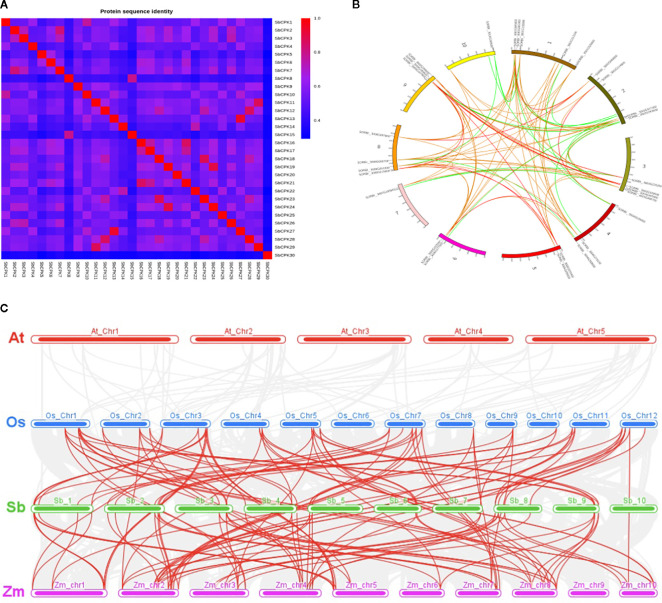
Analysis of similarity and collinearity of *SbCPK* family genes. **(A)** Protein sequence identity within the SbCPK family. The color gradient from blue to red indicates the degree of sequence identity from low to high. **(B)** Chromosomal distribution and collinearity analysis of *SbCPK* genes. The outermost circular ring represents the gene density on the chromosome. The middle circle indicates the GC content of the chromosome nucleotide sequence. The innermost ring shows the chromosome name and its length. **(C)** Collinearity analysis of *CPK* family genes among *A. thaliana* (At), *O. sativa* (Os), *S. bicolor* (Sb), and *Z. mays* (Zm). The red lines highlight the syntenic *CPK* gene pairs.

Gene duplication is a major driving force of species evolution, with segmental and tandem duplications representing the primary mechanisms underlying gene family expansion (Zhao et al., 2025). Here, the evolutionary relationships of the *SbCPK* gene family were explored through sequence identity and collinearity analyses. Protein sequence identity among SbCPK members ranged from 29.2% to 100% (**Figure 4A**). Specifically, SbCPK13 and SbCPK27 shared the highest identity (93.5%), whereas SbCPK22 and SbCPK30 exhibited the lowest identity (29.2%).

Collinearity analysis identified multiple collinear *SbCPK* gene pairs in the sorghum genome, supporting their derivation from common ancestral genes. These paralogous pairs displayed varied degrees of sequence conservation, with *SbCPK18/SbCPK23* (92.8%), *SbCPK13/SbCPK17* (91.4%), and *SbCPK11/SbCPK29* (89.4%) showing high sequence similarity, indicative of relatively recent duplication events (**Figure 4B**). In comparison, several gene pairs exhibited low identity, such as *SbCPK18/SbCPK30* (31.2%) and *SbCPK30/SbCPK24* (31.6%), which likely resulted from ancient duplication events accompanied by extensive sequence variation during long-term evolution (**Figure 4B**). Collectively, these results demonstrate that the *SbCPK* gene family has experienced frequent duplication events, predominantly tandem duplications, which have contributed greatly to the functional diversification of *SbCPK* genes in sorghum.

We further analyzed interspecific collinearity of *CPK* genes between sorghum and three representative plant species, including *A. thaliana* (At), *O. sativa* (Os) and *Z. mays* (Zm), and constructed a multi-species collinearity map (**Figure 4C**). The results revealed the highest number of collinear *CPK* gene pairs between *S. bicolor* and *Z. mays (*51 pairs), followed by *S. bicolor* and *O. sativa* (40 pairs), whereas no collinear homologs were detected between sorghum and Arabidopsis. Most *SbCPK* genes exhibited conserved collinear counterparts in rice and maize, indicating that these *CPK* genes originated prior to the speciation of gramineous crops and have remained highly conserved throughout monocot evolution. The greater number of collinear pairs between sorghum and maize further supports their closer phylogenetic affinity compared with sorghum and rice. the absence of collinear *CPK* orthologs between sorghum (monocot) and *Arabidopsis* (dicot) indicates extensive genomic rearrangement during the long-term evolutionary differentiation of monocots and dicots, which has led to the loss of collinear conservation in the *CPK* gene family. Collectively, these findings demonstrate that the *CPK* gene family exhibits strong evolutionary conservation among monocot species. The retained collinearity of core *CPK* genes after species divergence suggests their essential and conserved biological functions in sorghum growth, development, and stress adaptation.

### 3.5 *Cis*-element analysis of the *SbCPK* gene family

**Figure 5:**
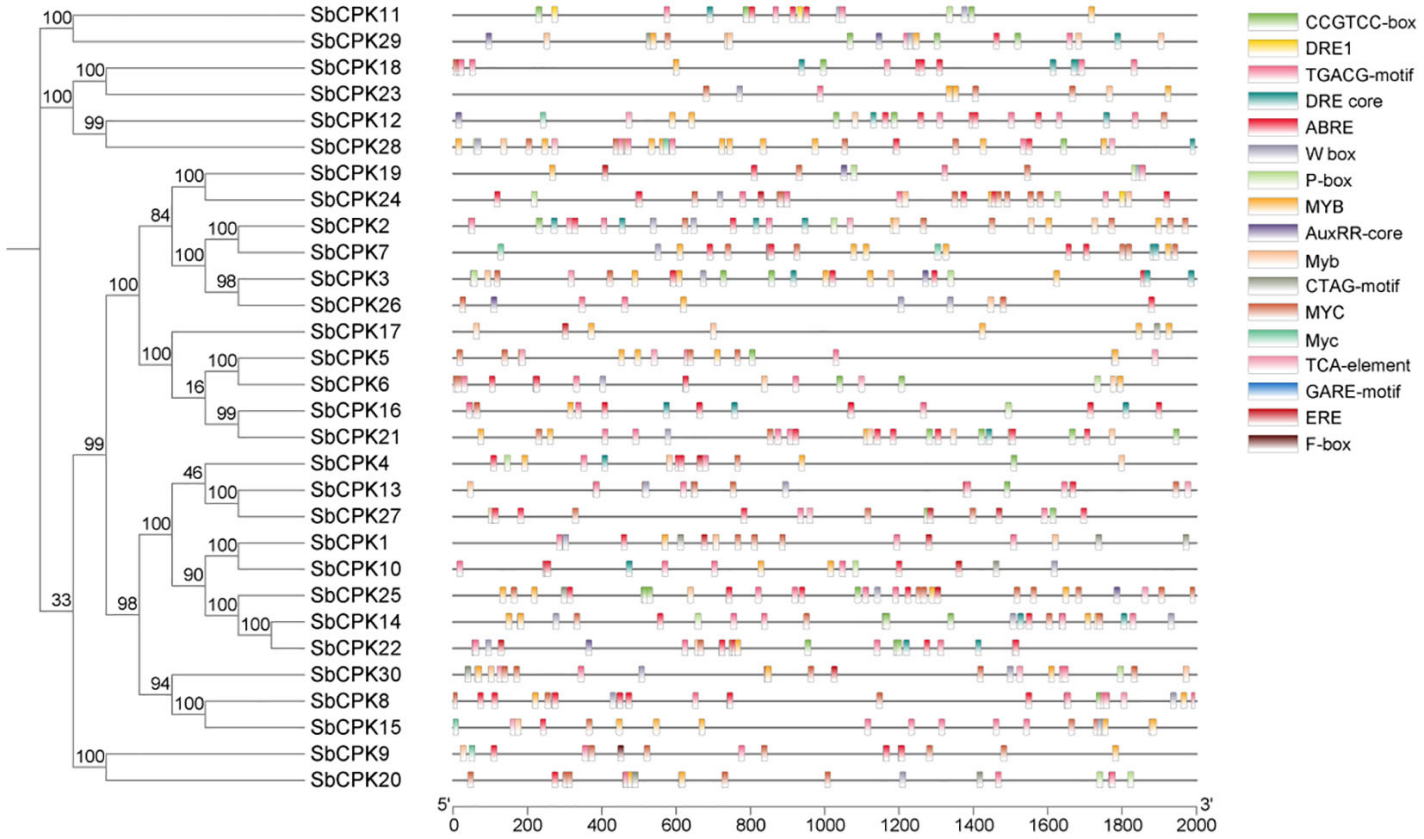
*Cis*-element analysis of *SbCPKs* promoters.

*Cis*-element analysis enables the prediction of probable regulatory associations and biological pathways involving target genes. To investigate the *cis*-regulatory structures of *SbCPK* promoters, the 2000 bp region upstream of the translational start of each *SbCPK* gene was analyzed using PlantCARE. The identified *cis*-elements were classified and statistically divided into three categories: abiotic and biotic stress response, plant hormone response, and plant growth and development (**Figure 5**; **Supplementary Table 3**).

The promoters of this gene family harbor abundant *cis*-elements, which exert potentially crucial functions in regulating gene expression. The promoters of the *SbCPK* gene family contain 29 light-responsive elements, such as A-box, AE-box, and ATC-motif; 14 hormone-responsive elements, such as ABRE (ABA-responsive elements), TCA-motif, and P-box; 16 stress-responsive elements (such as ARE and LTR), and 22 growth and development-related response elements (such as CAAT-box and CAG-motif). We noticed that the ABA-responsive ABRE element is widely distributed in the promoters of all *SbCPK* members except *SbCPK5* and *SbCPK23*. By contrast, the ethylene-responsive motif ERE is predominantly present in the promoter regions of *SbCPK1*, *SbCPK4*, *SbCPK30*, *SbCPK25* and *SbCPK10*. These patterns suggest potentially divergent expression profiles of *SbCPK* paralogs when responding to ABA and ethylene signals (**Figure 5**; **Supplementary Table 3**). Moreover, distinct *SbCPK* genes are potentially regulated by different transcription-factor families. For instance, the promoters of *SbCPK11*, *SbCPK2*5, *SbCPK28* and *SbCPK29* harbor W-box, which are specific binding sites for WRKY transcription factors. Meanwhile, the promoters of *SbCPK2*, *SbCPK*3 and *SbCPK7* contain MYB-binding motifs. These lines of evidence indicate that *SbCPK* genes may engage in various biological processes through multiple transcriptional regulatory cascades (**Figure 5**; **Supplementary Table 3**).

### 3.6 Effects of exogenous ABA and ACC treatments on sorghum seed germination

**Figure 6:**
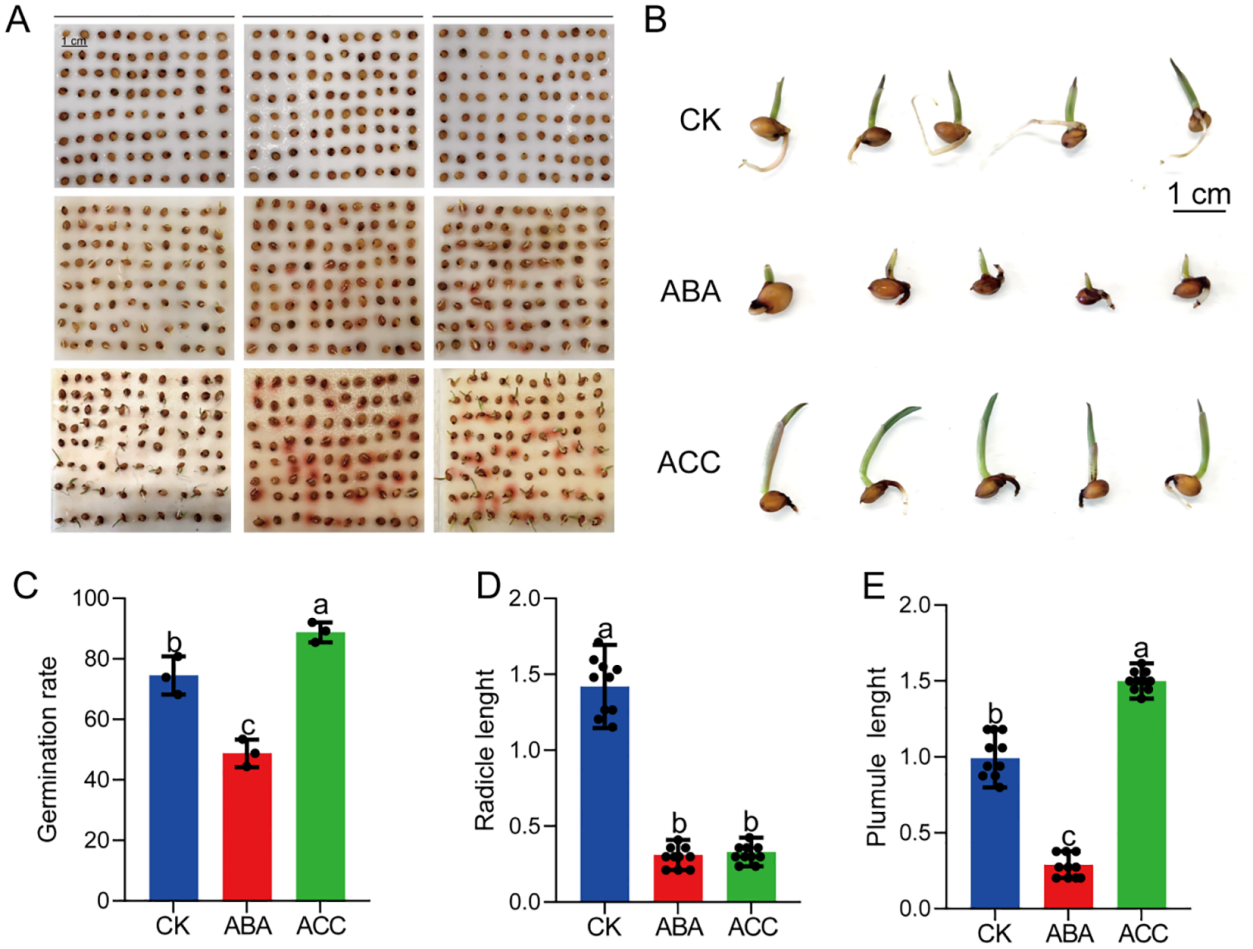
Germination phenotypes of sorghum seeds under ABA and ACC treatments. **(A)** Phenotypic observation of sorghum seeds at 0, 48 and 72 h after ABA and ACC treatments. **(B)** Seedling growth status at 72 h post hormone treatment. **(C)** Statistical analysis of seed germination rates after 72 h of treatment. **(D)** Statistical analysis of radicle length after 72 h of treatment. **(E)** Statistical analysis of plumule length after 72 h of treatment. Error bars represent standard deviation (SD). Germination rates were determined across three independent biological replicates, while radicle and plumule lengths were measured using 10 biological replicates each. One-way ANOVA was used for multi-group statistical comparisons, and different lowercase letters indicate significant differences (p < 0.05).

Our results demonstrated that exogenous ABA drastically suppressed germination of sorghum seeds, while ACC stimulated seed germination and embryonic growth (**Figure 6**). Germination rate, plumule length and radicle length of all treatment groups were quantified and statistically compared at 72 h post-hormone application. The control group exhibited an average germination rate of 74.5%; the ABA-treated group reached only 48.75%, whereas the ACC-treated group achieved 88.75% (**Figures 6A, C**). These data confirm that ACC significantly improves sorghum seed germination capacity, whereas ABA imposes strong inhibitory effects on germination. Statistical evaluation of radicle growth revealed that both ABA and ACC treatments significantly restrained radicle elongation relative to the control (**Figures 6B, D**). Measurements of plumule length further showed that 20 μM ACC markedly promoted plumule elongation, whereas 75 μM ABA drastically repressed plumule development (**Figures 6B, E**). Taken together, these observations illustrate divergent regulatory roles of ABA and ACC during sorghum seed germination and post-germination seedling growth. ABA negatively modulates seed germination as well as plumule and radicle development. By contrast, ACC enhances seed germination and plumule elongation yet represses radicle elongation.

### 3.7 Hormone-responsive expression patterns of *SbCPK* genes during seed germination

**Figure 7:**
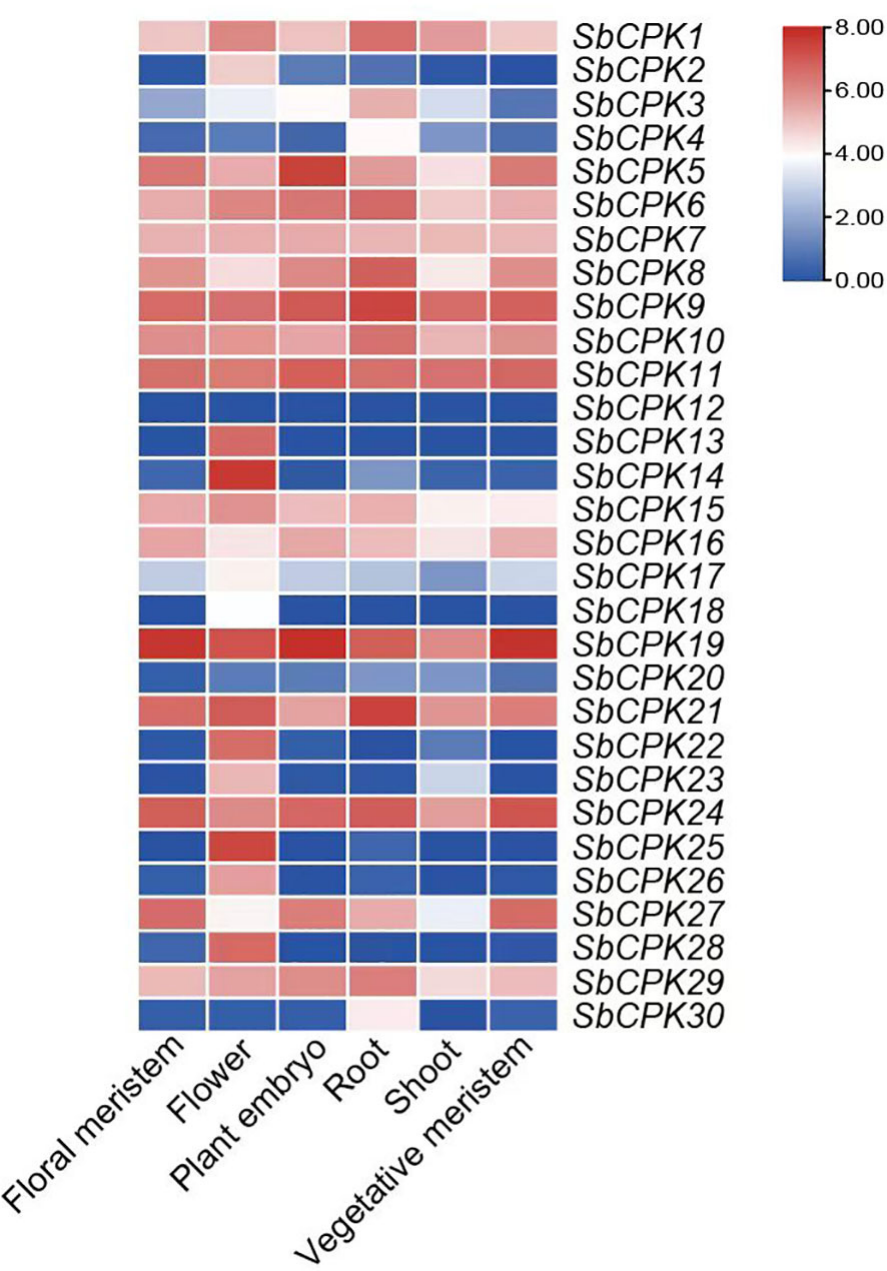
Expression patterns of *SbCPK* genes in different tissues of *S. bicolor*. The expression levels of the *SbCPK* gene family were displayed based on TPM-standardized values.

**Figure 8:**
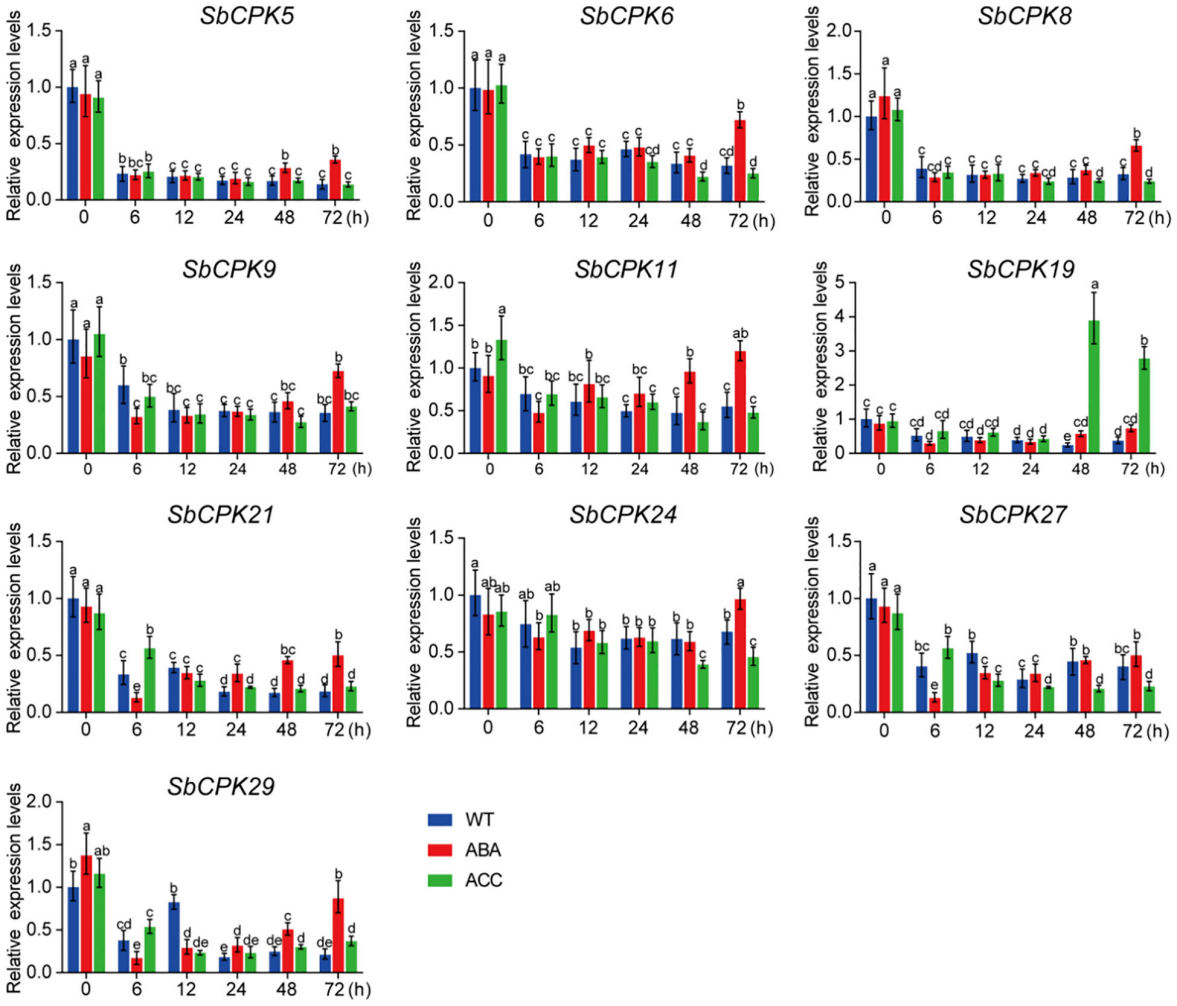
Expression profiles of *SbCPK* genes in response to ACC and ABA during sorghum seed germination. The expression levels of each *SbCPK* gene were quantified by qRT-PCR. The expression level for each gene in the CK plants at 0 h was normalized to 1.0. All experiments were conducted in triplicate with at least three independent biological replicates. Error bars indicate the SD of three biological replicates. One-way ANOVA was performed for statistical comparisons among multiple groups, and different lowercase letters denote significant differences at p < 0.05.

Public transcriptomic data was preliminarily analyzed to characterize the tissue-specific expression profiles of *SbCPK* genes (**Figure 7**). Several *SbCPK* genes exhibited widespread expression across multiple tissues: *SbCPK5*, *SbCPK11*, *SbCPK19*, and *SbCPK24* were highly transcribed in embryos and roots. By contrast, *SbCPK12* showed nearly undetectable expression in all tested tissues, while *SbCPK13* and *SbCPK18* displayed tissue-specific expression restricted to flowers or embryos. The top ten *SbCPK* genes with the highest embryonic expression levels were selected for subsequent hormonal response analysis. Ranked by expression abundance, these genes were *SbCPK19*, *SbCPK5*, *SbCPK9*, *SbCPK11*, *SbCPK24*, *SbCPK6*, *SbCPK27*, *SbCPK8*, *SbCPK29* and *SbCPK21* (**Table 3**). Gene-specific primers were designed to investigate their dynamic expression patterns in germinating sorghum seeds under ABA and ACC treatments at different time points. Under ABA treatment, multiple *SbCPK* genes exhibited significant transcriptional upregulation. *SbCPK6*, *SbCPK8* and *SbCPK9* were markedly induced at 72 h, *SbCPK11* was upregulated at 48 h and *SbCPK21* showed an early upregulation at 24 h. Additionally, *SbCPK21* and *SbCPK29* shared a consistent expression trend, with expression initially repressed and subsequently activated (**Figure 8**). Under ACC treatment, *SbCPK19* expression was significantly elevated by 3-4 fold at 48 h, whereas *SbCPK8*, *SbCPK24*, *SbCPK27*, and *SbCPK29* displayed varying degrees of transcriptional repression.

**Table 3:** TPM standardized values of *SbCPK* genes.

Gene name	Floral meristem	Flower	Plant embryo	Root	Shoot	Vegetative meristem
SbCPK1	30	67	31	96	51	29
SbCPK2	0.1	27	1	0.7	0.1	0
SbCPK3	3	11	15	40	8	0.8
SbCPK4	0.5	1	0.4	15	2	0.6
SbCPK5	85	41	183	52	21	82
SbCPK6	41	69	86	103	28	40
SbCPK7	38	40	42	37	35	36
SbCPK8	58	22	66	120	19	61
SbCPK9	100	94	129	170	98	116
SbCPK10	62	56	46	92	37	60
SbCPK11	94	81	115	94	90	106
SbCPK12	0	0	0	0	0	0
SbCPK13	0	103	0	0	0	0
SbCPK14	0.4	200	0.1	2	0.3	0.3
SbCPK15	43	59	33	39	17	18
SbCPK16	46	20	44	33	20	40
SbCPK17	6	17	6	5	2	7
SbCPK18	0	14	0	0	0	0
SbCPK19	214	139	230	120	64	224
SbCPK20	0.2	1	1	2	2	0.7
SbCPK21	100	125	48	180	57	77
SbCPK22	0.1	98	0.2	0	1	0
SbCPK23	0	36	0.1	0.1	7	0
SbCPK24	119	65	107	125	50	137
SbCPK25	0	166	0	0.4	0	0
SbCPK26	0.2	49	0	0.3	0	0.1
SbCPK27	99	16	77	41	11	101
SbCPK28	0.4	104	0	0	0	0.1
SbCPK29	35	48	62	75	23	33
SbCPK30	0.2	0.2	0.2	18	0	0.3

## 4 Discussion

### 4.1 The evolutionary and structural characteristics of the SbCPK gene family support its involvement in the regulation of seed germination

In this study, 30 *SbCPK* genes were systematically identified based on the sorghum BTx623 reference genome. These genes are asymmetrically distributed across 10 chromosomes and form 8 tandem duplication clusters, demonstrating that tandem and segmental duplications serve as the major evolutionary drivers underlying SbCPK family expansion. This evolutionary pattern is highly consistent with that of CPK families in other gramineous crops, including wheat, maize and rice (Khalid et al., 2019; Liu et al., 2023; Mittal et al., 2017). Phylogenetic analysis categorized *SbCPKs* into four subfamilies, which clustered closely with CPK homologs from Arabidopsis, rice and maize. These findings suggest that the CPK family has undergone ancient evolutionary divergence prior to the differentiation of monocots and dicots, and core functional modules associated with seed development and germination have been conserved in gramineous crops (Cheng et al., 2002; Ray et al., 2007; Asano et al., 2011).

Gene structure and conserved motif analyses revealed that all SbCPK proteins harbor canonical serine/threonine kinase domains and EF-hand calcium-binding motifs. Members within the same subfamily exhibit highly conserved exon-intron architectures, which provides a solid structural foundation for their biological functions as calcium-dependent protein kinases (Ormancey et al., 2017). Subcellular localization predictions revealed that SbCPK proteins are likely distributed in multiple subcellular compartments, including the cytoplasm, chloroplasts, mitochondria and endoplasmic reticulum, implying functional divergence among members of this gene family. For instance, Arabidopsis AtCPK4 and AtCPK11 are localized in both the cytoplasm and nucleus, and these two functionally redundant kinases positively mediate ABA-induced repression of seed germination (Zhu et al., 2007). In maize, ZmCPK15, ZmCPK36 and ZmCPK38 are all plasma membrane-localized and capable of physically interacting with PP2C family proteins (Khalid et al., 2019). N-terminal myristoylation and palmitoylation modifications are well-established regulators governing the subcellular localization of CPK proteins. Myristoylation occurs at the Gly2. Prior myristoylation of Gly2 serves as a prerequisite for subsequent palmitoylation of adjacent N-terminal cysteine (Cys) residues. Myristoylation alone only mediates weak and transient protein-membrane interactions, whereas combined palmitoylation enables stable anchoring of CPK proteins onto the plasma membrane or endoplasmic reticulum membrane, ultimately determining their subcellular distribution (Martín and Busconi, 2000; Lu and Hrabak, 2013; Lima-Huanca et al., 2025). In this study, nearly all sorghum SbCPK isoforms harbor a conserved second-position N-terminal glycine residue, except SbCPK17, SbCPK19, SbCPK22, SbCPK24 and SbCPK30, which implies their potential membrane-targeting capacity (**Supplementary Table 1**). For instance, rice OsCPK2 was the first identified CPK validated to undergo N-terminal dual acylation. Both the Gly2 myristoylation site and the Cys4 palmitoylation site are indispensable. A G2A (The glycine-to-alanine substitution at position 2) abolishes myristoylation. Even with an intact Cys palmitoylation site, the mutant protein loses plasma-membrane localization and disperses within the cytosol (Martín and Busconi, 2000). In Arabidopsis, the first 16 N-terminal amino-acid residues of AtCPK5 are sufficient to direct plasma-membrane targeting. A G2A-mediated disruption of myristoylation triggers dissociation of AtCPK5 from the plasma membrane and results in cytosolic diffusion (Lu and Hrabak, 2013). Future experiments are therefore required to characterize the myristoylation- and palmitoylation-mediated post-translational modifications as well as their effects on the subcellular trafficking of sorghum SbCPK proteins.

Analysis of promoter *cis*-acting elements revealed abundant ABREs, EREs, seed-specific regulatory elements, and MYB/MYC binding sites in the promoter regions of SbCPK genes. These predicted *cis*-acting motifs suggest that *SbCPK* genes may potentially integrate hormone signals and developmental cues, thereby participating in the regulatory modulation of seed germination (Lescot et al., 2002; Liu et al., 2023). Interspecies collinearity analysis showed that sorghum shares numerous CPK collinear gene pairs with maize and rice; these collinear genes have been shown to participate in regulating seed germination in maize and rice, further supporting the conserved function of *SbCPK* genes in sorghum seed germination (Wei et al., 2014; Ray et al., 2007; Ding et al., 2013).

### 4.2 Expression patterns of *SbCPK* genes in response to ABA and ethylene during seed germination

Seed germination is a sophisticated biological process fine-tuned by the antagonistic regulatory relationship between germination-inhibiting ABA and germination-promoting ethylene, with calcium signaling serving as a core mediator of their signaling crosstalk (Finkelstein et al., 2008; Benech-Arnold and Rodriguez, 2018; Li et al., 2025). Tissue-specific expression profiling in this study demonstrated that *SbCPK5*, *SbCPK9*, *SbCPK11*, and *SbCPK19* were specifically and highly expressed in embryos, suggesting their potential roles in modulating seed germination. This expression pattern is consistent with previously characterized CPK genes in Arabidopsis (*AtCPK2*, *AtCPK11*) and rice (*OsCPK2*), which exhibit embryonic-specific expression and participate in the regulation of seed germination (Liang et al., 2025; Kilburn et al., 2022; Morello et al., 2000). Accumulating evidence has revealed that plant CPK family proteins participate in grain development by modulating starch metabolism, amylose accumulation, and endosperm transparency (Jiang et al., 2018). As pivotal calcium sensors, CPKs also govern seed dormancy and germination through the mediation of phytohormone signaling cascades. For instance, Arabidopsis *AtCPK12* represses ABA signaling during seed germination and early seedling growth (Ali et al., 2022; Zhao et al., 2011), while wheat *TaCPK40* negatively regulates seed dormancy by modulating ABA sensitivity and thereby facilitates seed germination (Liu et al., 2023).

Exogenous hormone treatment verified that 75 μM ABA significantly inhibited sorghum seed germination, as well as plumule and radicle elongation, while 20 μM ACC markedly promoted germination. These results are consistent with canonical regulatory effects of ABA and ethylene on seed germination reported in previous studies (Roberto et al., 2018; Rodríguez et al., 2021; Ju et al., 2025). qRT-PCR analysis revealed that most *SbCPK* genes substantially upregulated in response to ABA treatment. Among them, *SbCPK6*, *SbCPK8*, *SbCPK9*, *SbCPK11*, and *SbCPK21* exhibited significantly elevated expression, implying that these genes positively regulate ABA signaling to suppress seed germination. It is well documented that CPKs enhance ABA signaling via phosphorylation of core transcription factors such as ABF/ABI5, thereby repressing seed germination (Choi et al., 2005; Zhang et al., 2020). In Arabidopsis, *AtCPK32* strengthens ABA signaling by phosphorylating ABF4 to inhibit seed germination (Choi et al., 2005). In wheat, *TaCPK2* enhances ABA-mediated germination inhibition by promoting ABI5 expression and stabilizing ABI5 protein (Liu et al., 2025). We hypothesize that ABA-inducible *SbCPK* genes in sorghum may activate downstream ABA signaling through conserved phosphorylation mechanisms to establish and maintain seed dormancy.

In contrast, ACC treatment significantly suppressed the expression of *SbCPK5*, *SbCPK24*, *SbCPK27* and *SbCPK29*, while the expression of *SbCPK19* was up-regulated by 3–4 fold at 48 h after ACC treatment. These distinct expression patterns imply potential functional divergence among SbCPK family members during ethylene signal transduction. Zhang et al. (2020) demonstrated that Arabidopsis *CPKs* regulate ethylene-mediated seed germination via phosphorylation of EIN3, a core ethylene signaling regulator. Based on our transcriptomic profiling data, we hypothesize that ACC-inducible *SbCPK19* may mediate ethylene signaling to facilitate sorghum seed germination, whereas the ACC-repressed *SbCPKs* potentially act as negative regulators of this germination process. This proposed regulatory framework requires further biochemical and genetic validation for confirmation.

### 4.3 Hypothetical regulatory model of *SbCPK* genes in seed germination and their prospective implications for PHS tolerance breeding

PHS, which occurs as premature grain germination under high temperature and high humidity field conditions, severely restricts sorghum yield and processing quality (Rodríguez et al., 2021). Elucidating the molecular regulatory mechanisms underlying seed dormancy and germination is essential for exploring potential genetic strategies to improve PHS tolerance in cereal crops. The qRT-PCR results suggest that the *SbCPK* family may participate in calcium signal-mediated crosstalk between ABA and ethylene during sorghum seed germination, implying their potential involvement in the modulation of seed germination behaviors associated with PHS-related physiological traits.

Integrating the current expression profiling data and previously reported plant hormone regulatory pathways, we herein propose a hypothetical preliminary mechanistic model for SbCPK-mediated regulation of sorghum seed germination. This model remains speculative and requires further functional verification. Briefly, during seed dormancy, ABA accumulation may trigger the upregulated expression of calcium-dependent protein kinases, including *SbCPK6*, *SbCPK9*, and *SbCPK11*. These SbCPK members are hypothesized to potentially reinforce downstream ABA signaling by phosphorylating ABF/ABI5 transcription factors, thereby contributing to the maintenance of seed dormancy. In contrast, during seed germination, elevated ethylene signaling may specifically induce the expression of *SbCPK19* to activate ethylene-dependent cascades, while repressing the expression of ABA-responsive *SbCPK* genes (e.g., *SbCPK5* and *SbCPK24)*. Such transcriptional antagonism between ABA- and ethylene-associated SbCPK responses is speculated to facilitate seed germination progression.

Based on the distinct hormone-responsive expression patterns observed in this study, *SbCPK19*, *SbCPK9*, and *SbCPK11* represent potential candidate genetic resources that may be associated with the modulation of seed germination characteristics relevant to PHS tolerance. It is speculated that genetic modification of these genes (e.g., knockout of *SbCPK19* or overexpression of *SbCPK9/SbCPK11*) potentially optimize seed dormancy performance and further improve PHS tolerance in sorghum, which requires future genetic validation. Notably, the current study has certain limitations. All conclusions are inferred solely from transcript expression profiles, and no genetic functional verification, kinase activity detection, or protein interaction experiments were performed. The proposed regulatory model is merely a preliminary hypothesis rather than a confirmed molecular mechanism. Further systematic experiments, including CRISPR/Cas9 gene editing, *in vitro* kinase assays, and yeast two-hybrid screening, are necessary to verify the biological functions of SbCPK proteins, identify their downstream interacting substrates, and clarify their precise regulatory networks. These follow-up studies will help consolidate the theoretical basis for utilizing *SbCPK* genes in sorghum molecular breeding targeting PHS tolerance.

## 5 Conclusions

Based on the sorghum BTx623 v2.1 reference genome, this study performed a full-scale genome-wide identification and systematic analysis of the *SbCPK* family. The evolutionary patterns, structural conservation, and hormone- and stress-related *cis*-regulatory elements of the 30 identified *SbCPK* genes were comprehensively characterized, which enriches the fundamental genomic information of the sorghum SbCPK family and provides important data support for subsequent functional characterization of *CPK* genes in gramineous crops. The qRT-PCR validation revealed that *SbCPK* family genes exhibit distinct tissue-specific expression profiles and display divergent response patterns to ABA and ethylene signals during seed germination. Several core *SbCPK* with embryo-specific high expression were identified, indicating a potential association of this gene family with the hormonal regulation of sorghum seed germination. This study screened a set of candidate genes closely related to seed germination in sorghum. Among them, *SbCPK19*, *SbCPK9*, and *SbCPK11* represent promising targets for exploring the calcium signaling-mediated regulation of PHS, providing valuable genetic resources for molecular breeding of sorghum. Collectively, this study preliminarily elucidates the response mode of the Ca²^+^-CPK signaling pathway to ABA and ethylene during sorghum seed germination, establishes a preliminary theoretical basis for uncovering the molecular mechanisms underlying sorghum seed germination, and offers a novel insight into the molecular improvement of PHS tolerance in gramineous crops.

## Data Availability

The datasets presented in this study can be found in online repositories. The names of the repository/repositories and accession number(s) can be found in the article/**Supplementary Material**.
