# Supplementary material for: Genome-wide analysis and expression profiling of CPK gene families in Sorghum bicolor (L.) Moench reveal their hormone-mediated responses during seed germination
Source: Front Plant Sci. 2026 Jul 29;17:1897022. doi: 10.3389/fpls.2026.1897022 (PMC13462744; doi:10.3389/fpls.2026.1897022)
Supplement: Supplementary file 1 [file Image1.pdf]

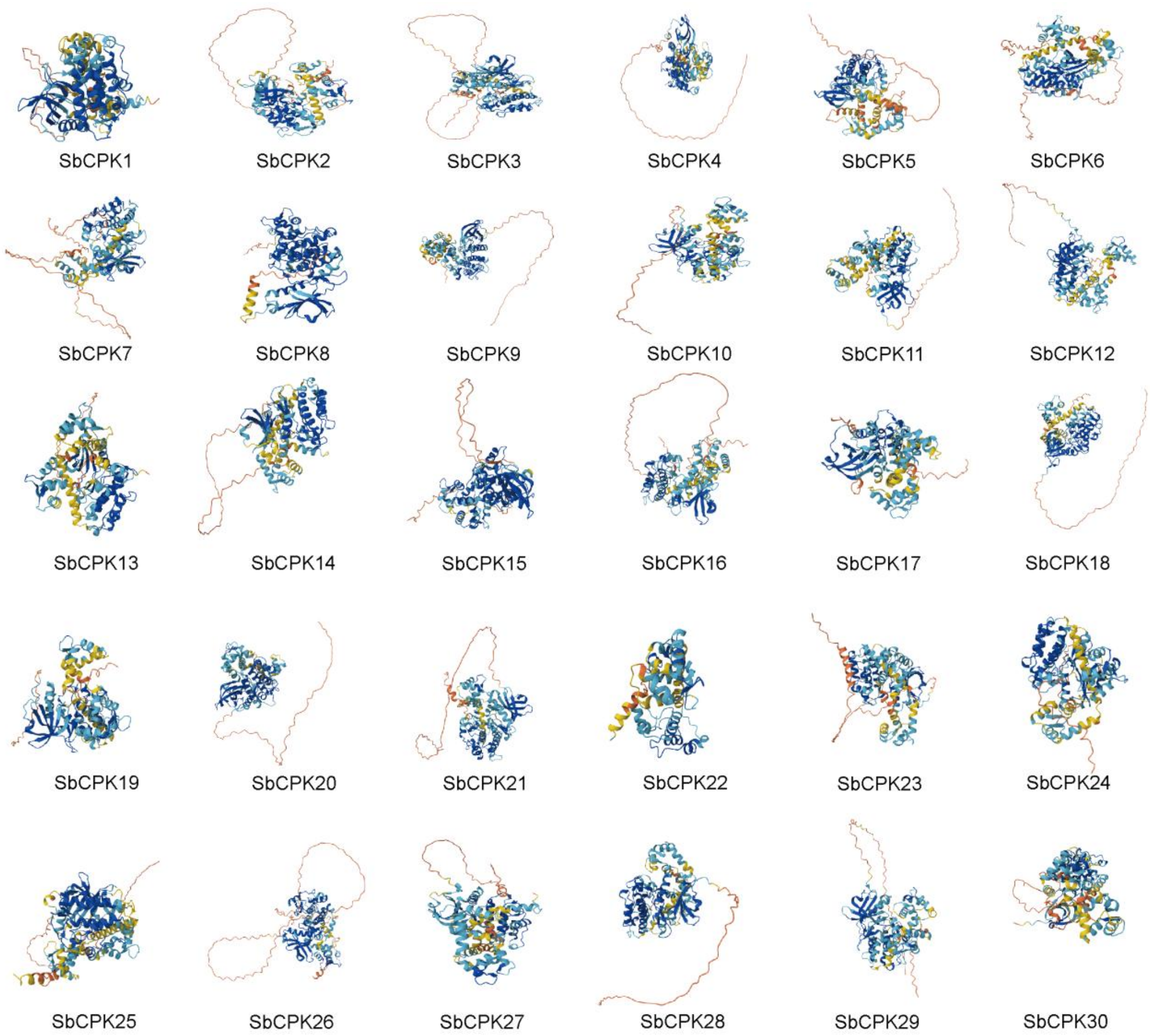

Supplemental figure 1. Predicted three-dimensional (3D) protein structures of SbCPK family members, showing conserved spatial conformation across the gene family.
